# Neutrophils in the hepatocellular carcinoma microenvironment: orchestrators of progression and immunity

**DOI:** 10.3389/fimmu.2026.1735189

**Published:** 2026-02-03

**Authors:** Yanjie Lian, Li Wang, Jiuchong Wang, Dan Zhu, Wenliang Lyu

**Affiliations:** Guang’anmen Hospital of the Chinese Academy of Traditional Chinese Medicine, Beijing, China

**Keywords:** hepatocellular carcinoma (HCC), neutrophil, neutrophil extracellular traps (NETs), tumor microenvironment(TME), tumor-associated neutrophils (TANs)

## Abstract

Among all malignant tumors, liver cancer is highly common, and hepatocellular carcinoma (HCC) stands as its most frequently seen pathological form. The majority of HCC patients are difficult to be detected or treated at an early stage. Concurrently, the postoperative recurrence rate remains relatively high, leading to a poor clinical prognosis of HCC. Recently, immunotherapy has made it promising to treat HCC. tumor microenvironment (TME) matters considerably in HCCprogression and metastasis. Neutrophils belong to the innate immune system’s essential elements, and their role as key regulators in the HCC-TME is becoming more widely recognized. By studying neutrophils ‘ pro-tumor and anti-tumor mechanisms in HCC, it is expected to gain a deeper comprehension of the functions of neutrophils and further reveal their biological characteristics. In addition, we analyze the crosstalk between neutrophils and other cellular constituents of the TME, and discuss emerging therapeutic strategies that target neutrophil-centric pathways. A deeper understanding of neutrophil biology will both illuminate the complexity of immune networks in liver cancer and offer a theoretical framework for HCC prevention and treatment.

## 1 Introduction

Liver cancer poses a major global health burden. Data from the WHO’s International Agency for Research on Cancer shows that in 2022, there were around 870,000 new liver cancer cases and some 760,000 related deaths worldwide (1). It currently ranks as the third leading cause of cancer-related mortality globally, with a five-year relative survival rate of roughly 18% (2), and its incidence is expected to increase in the future. Hepatocellular Carcinoma (HCC) is the most prevalent histopathological subtype of primary liver cancer, representing approximately 85% of all cases (3). HCC is a highly aggressive malignancy characterized by rapid progression, limited therapeutic options, and a poor overall prognosis. Currently, several established clinical treatment modalities, including interventional therapies, surgical resection, liver transplantation, chemotherapy, radiotherapy, and molecular targeted therapies, are available for HCC. However, due to its frequently asymptomatic early phase, HCC is frequently diagnosed at intermediate or advanced stages. Furthermore, tumor recurrence, drug resistance, and metastasis remain key hurdles that impede the effective management of the disease. These limitations underscore the importance of comprehensive treatment strategies, particularly immunotherapy, in improving patient outcomes.

In recent years, as cancer research has shifted from focusing on cancer cells to the entire tumor ecosystem, attention has been drawn to the tumor microenvironment (TME), offering an innovative approach for the search of novel treatment approaches (4) inflammation is tied to approximately 90% of HCC cases, emerging as a pivotal trigger in hepatocarcinogenesis (5, 6). Studies have shown that chronic inflammation drives carcinogenesis by directly inducing gene mutations in hepatocytes (7), while promoting tumor invasion, proliferation, and metastasis by constructing a TME (8). HCC-TME includes two parts: cellular components and their non-cellular counterparts. Among them, the former include tumor-associated neutrophils (TANs), tumor-associated macrophages (TAMs), hepatic stellate cells (HSCs) and cancer-associated fibroblasts (CAFs), etc. The latter, consisting of various extracellular matrices (ECMs), growth factors, inflammatory cytokines, etc., which matter significantly in developing HCC (9, 10)-oncology is undergoing a paradigm shift from “adaptive immunity” (e.g., PD-1 inhibitors) to “innate immunity reprogramming” (11). Over the past decade, neutrophils, boasting their dual role in cancer biology, have attracted considerable attention and are increasingly recognized as critical TME components.Research has demonstrated that neutrophils can actively migrate into the TME along chemotactic gradients established by tumor-secreted cytokines and subsequently differentiate into TANs under the influence of various cytokines present in the microenvironment (12).Moreover, it can manifest as a dual effect of promoting and suppressing tumors, and can transform into each other under certain conditions (13), it may be a breakthrough in microenvironmental regulation of HCC. However, the precise regulatory mechanisms as well as functional roles of neutrophils in HCC are still unclear. This article intends to thoroughly re-evaluate neutrophils ‘ significant role in the occurrence, development, as well as prognosis of HCC, expecting to better understand the immunological microenvironment of HCC and forge a theoretical foundation to improve the efficacy of immunotherapy for HCC and seek novel clinical treatment targets.

## 2 Biological characteristics and classification of neutrophils

### 2.1 Overview of the activation and function of neutrophils

Neutrophils originate from myeloid precursor cells in the bone marrow and represent the most abundant granulocyte population, comprising 50%–70% of circulating leukocytes in most mammalian species. These cells serve as critical primary effector cells in innate immune responses (14).Conventionally, neutrophils have been considered to primarily function in host defense, immune regulation, and tissue injury (15),with a short circulating half-life of 4–18 hours (16, 17).However, emerging evidence demonstrates that TANs exhibit significantly extended lifespans, surviving for more than 5 days within the tumor microenvironment (18). This prolonged survival allows TANs to produce and release increased quantities of bioactive molecules, thereby functionally contributing to tumor development and progression.

### 2.2 Neutrophil heterogeneity and tumorigenesis

#### 2.2.1 Circulating neutrophils’ phenotype and function in tumors

In accordance with neutrophils ‘ density gradient in the blood, they are classified into high-density neutrophil (HDN) as well as low-density neutrophil (LDN). LDN in circulation is an immature, banded or degranulated mature and senescent neutrophil released from the bone marrow, which has an immunosuppressive tumor-promoting effect, while HDN serves as the anti-tumor tool (19). During early cancer development, HDN is dominant and has an overall anti-tumor response. As cancer progresses, LDN becomes dominant, redirecting the neutrophil phenotype toward protumorigenic functions. In addition, according to cell density and surface markers, circulating neutrophils in cancer patients were divided into three subgroups: mature LDN, immature LDN and HDN. According to the terms of different polarizationstates of neutrophils, they are classified as polymorphonuclear granulocyte (PMNs), polymorphonuclear myeloid⁃derived suppressor cells (PMN⁃MDSCs), as well as LDNs and HDNs.

#### 2.2.2 Neutrophils’ phenotype and function in TME

Xue et al. (20) pioneered single-cell RNA sequencing analysis of neutrophils in the HCC-TME, demonstrating that neutrophil heterogeneity is reflected not only in phenotypic markers but also in functional properties. The plasticity and diversity of neutrophils form the basis for the dual potential of TANs within the tumor microenvironment (21). Similar to TAMs, TANs exhibit both pro-tumorigenic and anti-tumorigenic effects (22). In 2009, Fridlender et al. (22) proposed that TANs could be classified into tumor-inhibiting N1 and tumor-promoting N2 subtypes, analogous to the M1/M2 paradigm for TAMs. Studies have demonstrated that N1 TANs inhibit tumor progression through two primary mechanisms: direct cytotoxic killing of tumor cells and indirect activation of other immune cells. Conversely, N2 TANs promote tumor invasion, metastasis, and angiogenesis by releasing tumor-supporting factors and remodeling the extracellular matrix (23, 24). Therefore, preventing TAN conversion to the N2 phenotype has emerged as an important therapeutic strategy. Within the TME, neutrophils are exposed to multiple polarization signals. Tumor-derived transforming growth factor-β (TGF-β) and granulocyte colony-stimulating factor (G-CSF) are dominant drivers that promote N1→N2 conversion (25, 26). Conversely, type I interferon (IFN) signaling antagonizes this process and maintains or restores N1 polarization (27). Thus, therapeutic strategies targeting the TGF-β pathway aim to block N1→N2 conversion, while IFN-based approaches seek to actively promote N1 programming.

However, unlike the well-established M1/M2 TAM paradigm, the N1/N2 TAN classification lacks specific surface markers to clearly distinguish these subsets in tumors. The surface markers, transcriptional regulators, and cytokine profiles of N1/N2 TANs require further characterization (28). With the advancement of single-cell RNA sequencing(scRNA-seq), large-scale multi-omics integration, and mass cytometry technologies, the classification of TANs has evolved from the conventional N1/N2 binary paradigm toward a more nuanced, multi-state lineage model—including inflammatory/chemotactic, immunosuppressive,antigen-presenting cell (APC)-like, and NISG phenotypes et al. Concurrently, candidate markers based on surface molecules (e.g., OLR1, CXCR2, HLA-DR, CD74, LY6E) and transcription factors (e.g., BHLHE40, STAT1, IRF7/IRF9) have been increasingly identified, which lays the foundation for establishing a more accurate TAN classification system. Jaillon et al. (21) classified TANs into immature neutrophils (immature neutrophil) based on the differences in TANs phenotypes, there are four types: immature neutrophil(NI), anti-tumor type (N1 type), pro-tumor type (N2 type), and neutrophil with interferon-stimulated gene signature (NISG). Based on the summary of the current evidence, we have identified a number of functionally distinct TAN subsets that have unique molecular markers and mediate different tumor immune processes. (**Table 1**).

**Table 1:** TAN subtype classification and biomarker integration.

Subtype name	Core phenotype/function	Candidate surface markers	Recommended testing methods	Key literature
Inflammatory/Chemotactic	High expression of IL1β and CXCR2 promotes tumor metastasis	CXCR2^+^ IL1β^+^ CD11b^+^	scRNA-seq cluster CXCR2^+^IL1β^+^; Flow cytometry(FC): CXCR2-PE	Albrengue R, 2024 (29)
Immunosuppressive	MIF^+^OLR1^+^, Arg1↑, Inhibit T cells	OLR1^+^ MIF^+^CD11b^+^ Ly6G^+^	scRNA-seq:MIF^+^OLR1^+^ signature; FC: OLR1-APC	Zeng Y,2025 (30)
APC-like type	Strong antigen presentation,activation of CD8^+^T cells	HLA-DR^+^ CD74^+^CD11c^+^ CD80^+^	scRNA-seq:HLA-DRA^+^CD74^+^; FC: HLA-DR-BV421/CD74-FITC	Wu Y, 2024 (31)
NISG type(IFN-stimulated,Ly6E^hi)	Predict ICB response, ISG ↑	Ly6E^hi PD-L1^+^CD11b^+^	scRNA-seq:Ly6E^+^ISG signature(IFIT3/MX1); FC: Ly6E-BV711 scRNA-seq: VEGFA^+^	BenguiguiM, 2024 (32)
VEGFA^+^angiogenic type	VEGFA^+^, promote angiogenesis	VEGFA^+^ CXCR4^+^ CD66b^+^	cluster;IHC: VEGFA^+^CD66b^+^	Yang Y,2025 (33)

### 2.3 Dual role of neutrophil extracellular traps in HCC

The DNA-histprotein-granule protein complexes released after the activation of neutrophils is also known as NETs, and the formation process of nets is called NETosis. Initially found in the host defence against pathogen invasion, NETs have now been proven important in tumors ‘ occurrence and development (34). For one thing, by promoting tumor metastasis and thrombosis, NETs accelerate disease progression (35). For another, by reproducing the TME, NETs promote inflammation and various types of tumor cell proliferation, differentiation, transfer (36). Some studies also suggest that similar to the dual potential of TANs in tumor development,NETs also have dual effects of promoting and anti-tumor (37). Previous studies on NETs have mainly focused on their overall effects. However, recent studies indicate that the functional duality of NETs appears to be highly context-dependent. In non-HCC tumor models (e.g., pulmonary metastasis). MPO, proteases and histones, among other NETs components, can destroy tumor cells and prevent their development and metastasis (38) However, in established HCC, the overwhelming evidence supports a net pro-tumor effect. The DNA scaffold within NETs and associated HMGB1 activate the TLR9/NF-κB axis to promote HCC cell proliferation and metastasis (39). Similarly, neutrophil elastase (NE) drives angiogenesis by activating MMP-9 and degrading TSP-1 (39). Therefore, while theoretical anti-tumor effects exist, their clinical relevance in HCC is likely limited to highly specific temporal or spatial contexts that remain to be defined.

## 3 Neutrophils’ recruitment and communication in HCC-TME

**Figure 1:**
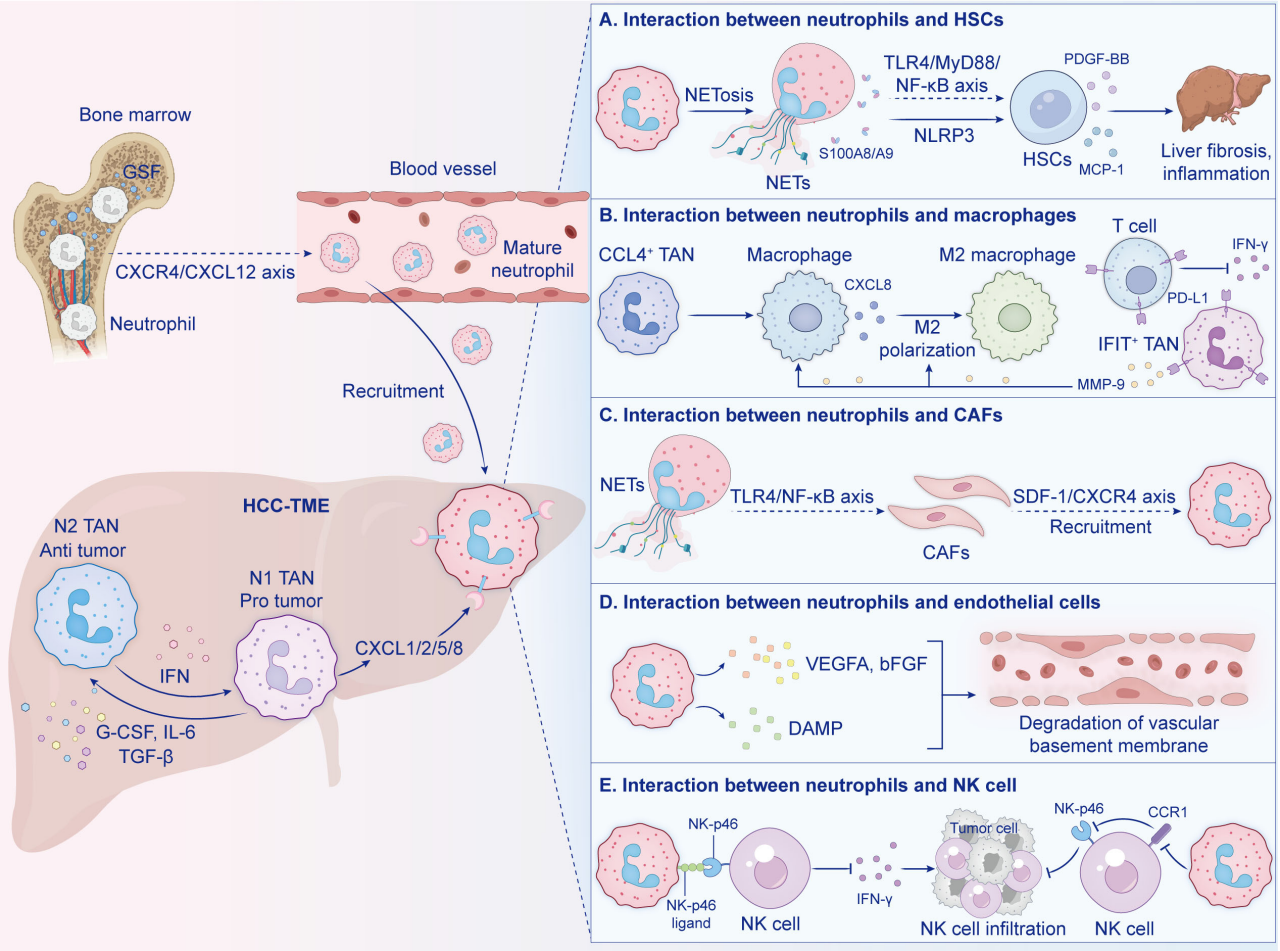
Neutrophils ‘ recruitment and communication in HCC-TME. In section **(A)** neutrophils and hepatic stellate cells (HSCs) interact via pathways leading to liver fibrosis. Section **(B)** shows neutrophil and macrophage interactions impacting polarization. Section **(C)** illustrates the interaction with cancer-associated fibroblasts (CAFs) influencing recruitment. Section **(D)** highlights interactions with endothelial cells affecting the vascular membrane. Section **(E)** depicts interactions with natural killer (NK) cells impacting tumor cells.

However, there are almost no neutrophils in a normal liver. Consequently, where do the neutrophils in HCC-TME come from? And how does it infiltrate into the liver? Generally, it is acknowledged that neutrophils ‘ migration from the bone marrow to the tumor site is divided into three stage (40): 1) expansion and maturation of premature neutrophils in the bone marrow; 2) circulation in the blood vessels by attaching to endothelial cells; and 3) chemotactic movement of neutrophils to the tumor site. In HCC-TME, neutrophils’ activation and recruitment are precisely regulated by multiple factors and pathways, and are the result of communication between different cells (**Figure 1**).

### 3.1 Mechanism of neutrophil recruitment mediated by chemokine axes

G-CSF and granulocyte-macrophage colony-stimulating factor (GM-CSF) play a key role in neutrophils ‘ proliferation and maturation (41). G-CSF represents a cytokine, which is produced by a variety of cells, such as macrophages, endothelial cells, as well as cancer cells. Beyond fostering neutrophils’ proliferation and maturation, G-CSF also regulates neutrophil migration (42). GM-CSF has a similar effect. Neutrophils release a variety of chemokines to the TME, including CXCL8, CXCL1, CXCL2, and CXCL5, which bind to their surface receptor CXCR2 and constitute the core pathway of neutrophil activation and recruitment. Within the bone marrow, neutrophil release is primarily dependent on the dynamic balance between the CXCR4 and CXCR2 signalling pathways, as well as their interacting ligands (43). Among them, the CXCR4/CXCL12 axis transmits bone marrow residence signals in a high-affinity and gradient-dependent manner, maintaining the retention of neutrophils in the medullary, while the CXCR2 axis dominates the directional migration of neutrophils to the tumor site (44). G-CSF stimulation triggers the release of mature neutrophils from the bone marrow into peripheral blood. These cells are subsequently recruited to the tumor region in a targeted manner, driven by the chemotactic activity of CXCR2 ligands such as CXCL1, CXCL2, CXCL5, and CXCL8 (45). In addition, G-CSF can also positively regulate the migration ability of neutrophils by reducing the expression levels of CXCR4 and its corresponding ligand CXCL12 (46). Through the synergistic effect of CXCR2 axis, CXCR4 axis, and G-CSF, a stable chemotactic gradient is formed in the tumor microenvironment, which actively guides peripheral blood neutrophils to infiltrate into the tumor. This process notably increases the number of TANs and prompts these cells to tend to polarize into the pro-tumor N2 phenotype, thereby improving tumor angiogenesis, inducing immunosuppression and enhancing the ability of distant metastasis, ultimately accelerating the malignant progression of tumors.

### 3.2 Neutrophil-mediated intercellular signal crosstalk in HCC-TME

In HCC-TME, neutrophils are not passive effector cells, but rather a dynamic and central hub for signal integration and scheduling. Through precise intercellular signal crosstalk, neutrophils are embedded in a complex cell interaction network, and dynamically communicate with HSCs, TAMs, natural killer (NK) cells, etc., to jointly shape the pro-tumor microenvironment, “weaving” these originally functionally diverse cells into a functionally coordinated tumor-promoting network. This network precisely regulates almost all the key malignant phenotypes of HCC in space and time, such as angiogenesis, invasion and metastasis, immune escape.

#### 3.2.1 Synergistic effect with HSCs and TAMs

Neutrophils build a powerful immunosuppressive network through alliance with immunosuppressive cells, and HSCs and TAMs serve as key effector cells in liver inflammation, and activated HSCs synthesize and secrete a large amount of ECMs, which is deposited in the liver tissue in excess, resulting in abnormal liver structure and/or function, leading to the occurrence of fibrosis. Hepatic fibrosis is considered to be an inevitable stage for most chronic liver diseases ‘ progression to liver cancer (47), so the HSCs and is considered the key factor driving HCC development (48). In alcoholic metabolism-associated steatohepatitis (MASH), NETs accelerate hepatic fibrosis progression by directly activating HSCs and monocytes via NLRP3 (49). Chang et al. (50) showed that S100A8/A9 derived from neutrophils can stimulate HSCs to produce MCP-1 and PDGF-BB through TLR4/MyD88/NF- κβ axis, thereby promoting myofibroblast migration and accelerating the progression of fatty liver injury and fibrosis. Single-cell sequencing analysis based on the tissues of HCC patients revealed (20) that neutrophils were mainly enriched in subpopulations with immunosuppressive characteristics. Among them, CCL4+TANs actively recruit immunosuppressive cells such as macrophages through the CCL4-CCR5 chemotactic axis to jointly maintain the microenvironment’s immunosuppressive state, while IFIT+TANs highly express programmed cell death ligand-1(PD-L1), combine to programmed death-1 (PD-1) on the surface of T cells, direct inhibition of cytotoxic T cells and the secretion of IFN-γ, and weaken the anti-tumor immunity. MMP-9 released by neutrophils can jointly promote the polarization of macrophages towards M2 TAMs by activating latent TGF-β (51).M2 TAMs are the main source of neutrophil chemokines such as CXCL8, which can continuously attract more neutrophils to infiltrate the TME (52). Neutrophils interact with macrophages, intensifying liver inflammation and further damaging the liver (53), which collectively shapes a profoundly immunosuppressive microenvironment and impairs the body’s anti-tumor immune response.

#### 3.2.2 Interaction with CAFs and endothelial cells

Extensive crosstalk between neutrophils and stromal cells is a key link in remodelling the tumor stroma and promoting malignant behaviour. CAFs and endothelial cells are the two main interactors. HCC usually occurs in the context of cirrhosis, which is always associated with an enrichment of activated fibroblasts that are owed to chronic inflammation (54).CAFs are a major component of the TME, providing physical support and secreting various proteins, such as ECMs and hepatocyte growth factor (HGF), which play important roles in the occurrence and development of tumors (55). Neutrophils are the main source of CAFs (56) and have been shown to play significant roles in CAF activation. Takesue et al. (57) demonstrated that NETs can activate HSCs/CAFs through the TLR4/NF- κβ axis. Notably, the recruitment of neutrophils precedes that of CAFs, suggesting their critical involvement in the early stage of establishing the liver metastasis microenvironment. Cheng et al. (58) founded that CAFs can recruit circulating neutrophils through the SDF 1a/CXCR 4 signalling pathway and induce their differentiation into PD-1+ neutrophils, serving as a “response amplifier” for maintaining the continuous infiltration of neutrophils. Thus, an interdependent “matrix modification alliance” can be formed to promote connective tissue proliferation and tumor sclerosis. In the interaction with endothelial cells, the structure of NETs itself can serve as a physical scaffold for endothelial cell migration, and the NE on them can directly promote endothelial cell proliferation. Correspondingly, the activated tumor endothelial cells provide a gateway for the vascular exudation of neutrophils by highly expressing adhesion molecules such as E-selectin and ICAM-1, completing the positive feedback cycle of promoting angiogenesis.

#### 3.2.3 Interaction with NK cells

In addition to the interaction with adaptive immune cells, the strong inhibition of innate immune cells, especially NK cells, by neutrophils is an important link in tumor immune escape. In HCC, the NKp46 ligand expressed by activated neutrophils can bind to the activating receptor NKp46 on the surface of NK cells, inhibiting the cytotoxicity of NK cells and the production of IFN-γ (58, 59), resulting in a significant reduction in NK cells infiltration, particularly in advanced-stage HCC (60). Neutrophils also alleviate NK cells infiltration by inhibiting CCR1 and down-regulating the expression of NKp46 and NKG2D (61). In conclusion, neutrophils inhibit the activity of NK cells to promote tumor cells to evade the cytotoxicity of NK cells (62).

### 3.3 Heterogeneity of cell interaction networks among different subtypes of HCC

The causes of HCC include alcohol-related liver disease (ALD), metabolic disorder-related steatohepatitis (MASH), viral hepatitis, and other hepatotoxic and liver cancer predisposing factors. It must be pointed out that the neutrophil-dominated cell interaction network has a significant etiological dependence. In nonalcoholic steatohepatitis associated hepatocellular carcinoma (NASH-HCC), against the backdrop of inflammation and fibrosis, neutrophil-HSCs are the core driving tumorigenesis. Zhou et al. (63) indicated that activated neutrophils could directly stimulate the activation of HSCs through ROS and other mechanisms, and that activated HSCs could enhance the survival time of neutrophils by producing granulocyte-macrophage GM-CSF and IL-15, thus aggravating liver inflammation. NETs reprogram the energy metabolism of HSCs through the TLR3-COX-2-PGE_2_ axis and can activate quiescent HSCs through TLR3 signaling, thereby creating an environment conducive to the initiation of HCC (64). In HBV-HCC, chronic inflammation driven by viral antigens makes the neutrophil-TAM/T cell axis more prominent (65). Two critical players in the immunosuppressive milieu of HBV-related HCC are CD8+ T cells and TAMs (66).Viral antigens and inflammatory factors (e.g., IL-8) continuously recruit neutrophils and monocytes. TAN-N1 indirectly regulatesthe recruitment and activation of CD8+ T cells by producing chemokines and pro-inflammatory cytokines such as CCL3, CCL9, CXCL10, TNF- α, and IL-12, thereby contributing to the restriction of tumor growth (67). Understanding this heterogeneity is crucial for developing etiological specific immunotherapy strategies.

## 4 Pro-tumor effect of neutrophils in HCC

### 4.1 Promoting angiogenesis

Angiogenesis, which is the formation of new blood vessels from the existing vascular system, represents a key process for the growth and metastasis of HCC and is regulated by pro-angiogenic factor groups and anti-angiogenic factor groups (68). Vascular endothelial growth factor (VEGF) stands out as the most effective angiogenic factor in tumor angiogenesis. Cyclooxygenase-2 (COX-2), an inducible enzyme that stimulates the overexpression of VEGF, is thought to play a major role in tumor angiogenesis and progression (69). COX-2 and its downstream product PGE_2_ are highly expressed in HCC, and targeted inhibition of COX-2/PGE_2_ axis can effectively inhibit HCC angiogenesis, invasion, and metastasis (70, 71). A bioinformatics analysis study revealed the key role of COX-2 in TME, and its high expression level was observed to be closely related to the deposition of neutrophils and poor response to immunotherapy (72). NETs serve as another important factor promoting angiogenesis. The DNA/histone scaffold in NETs can upregulate VEGFR2 through the NF- κβ pathway by activating the TLR4/9 receptors on endothelial cells, thereby enhancing their response to VEGF and drives angiogenesis (18, 73). MMP-9 is a gelatinase, which is stored in the tertiary granules of neutrophils (74), like “molecular scissors”, MMP-9 can degrade the ECMs and basement membrane surrounding the tumor and promote angiogenesis, which removes physical obstacles for the migration and invasion of liver cancer cells, and is the first step for them to leave the primary focus and infiltrate the surrounding tissues. The new blood vessels not only provide nutrition for the growth of the primary tumor, but also provide “entrance” for tumor cells to enter the circulatory system and occur distant metastasis. In addition, neutrophils can also promote tumor development by releasing tumor regulatory protein M (OSM, IL-6, etc.), inducing VEGF expression, promoting tumor angiogenesis and metastasis, and increasing cancer cell infiltration (75, 76).

### 4.2 Promote the proliferation, migration and invasion of tumor cells

In the process of HCC progression, neutrophils promote the proliferation, migration, and inasieness of tumor cells through a variety of direct and indirectmechanism. Firstly, matrix metalloproteinases (MMP-8, MMP-9) and cathepsin G (Cat-G)released by neutrophils can cleave key matrix components such as endothelial cadherin and fibronectin, resulting in impaired integrity of basement membrane and ECMs (77). This not only causes endothelial dysfunction and vascular inflammation, creating channels for tumor cells to invade blood vessels, but may also facilitates to circulate tumor cells in colonizing distant organs, which is the initial key step in the metastasis process. Secondly, NETs act as important carrying and signalling hubs, playing a pivotal role in HCC metastasis. HBV-induced S100A9 activates RAGE/TLR4-ROS signalling, leading to the generation of a large number of NETs, thereby promoting HCC growth and metastasis (78). Clinical studies (79, 80) indicates that the level of NETs in HCC patients is significantly increased, positively correlated with the potential for tumor metastasis and negatively correlated with the survival rate of patients. The mechanism of action of NETs is multifaceted: NETs can activate dormant tumor cells and induce the release of a large number of proinflammatory factors such as IL-6 and TNF-α in cancer tissues and adjacent tissues. These factors subsequently activate NF-κβ, MAPK, and other signalling pathways, forming a positive feedback loop that continuously amplifies proliferation and migration signals and improves the invasiveness of tumor cells (81, 82). The transmembrane protein CCDC25 can act as a NETs-DNA receptor on cancer cells to sense extracellular NETs and activate the ILK-β-Parvin pathway to promote tumor migration (83). In addition, YANG et al. (80) found that NETs can encapsulate HCC cells, enabling them to resist cytotoxicity and enhance their invasion ability. Concurrently, these cells induce inflammatory responses by activating the TLR4/9-COX2 signalling pathway, promoting tumor metastasis. All these indicated that NETs may act as a “postal package” in the process of distant metastasis of tumors. Another subset of circulating neutrophils, identified as poor prognostic factors for overall survival in HCC patients, promotes HCC progression via the p53 and STAT3 signalling pathways (84).

### 4.3 Neutrophil-mediated suppression of T cell function and remodelling of the immunosuppressive microenvironment

In the HCC-TME, neutrophils strongly inhibit the activation, proliferation, and effector functions of T cells through various direct and indirect mechanisms, thus emerging as a key builder of the immunosuppressive microenvironment. Its inhibitory effect is mainly manifested in the following three aspects:

#### 4.3.1 Directly inducing apoptosis and functional exhaustion of T cells

N2 TANs can release reactive oxygen species (ROS) and enzymes such as arginase 1 (Arg1), creating an inhibitory metabolic environment (22). On the one hand, high levels of ROS and NO produced by inducible nitric oxide synthase (iNOS) can directly damage T cells and induce their apoptosis (85); on the other, Arg1 promotes immunosuppressive TME by depleting arginine, which is essential for T cell proliferation (86). Furthermore, PD-1 derived from neutrophils can transmit inhibitory signals, leading to T cell dysfunction or even exhaustion (87), thereby promoting immune escape of tumors. Another study has shown that TANs can inhibit T cell activity by releasing peroxide lipids through ferroptosis (88).

#### 4.3.2 Driving the differentiation of regulatory T cells by forming NETs

CD4+ T cells play a key role in immune surveillance of cancer (89, 90), and Tregs undermine cancer immune surveillance by creating an immunosuppressive environment that promotes tumor cell survival. Velliou et al. (91) found that NETs can promote Treg differentiation by activating the Toll-like receptor 4 (TLR4) receptor in CD4+ T cells, thereby promoting HCC progression. However, the detailed regulatory pathways remain to be further studied. WANG et al. (92) revealed that NETs promote Treg differentiation through metabolic reprogramming of naive CD4+ T cells, then secrete inhibitory cytokines, consume IL-2 competitively, and further weaken the anti-tumor response of effector T cells.

#### 4.3.3 Synergistically constructing an immunosuppressive network driven by hypoxia

The hypoxic environment inside the tumor is an important driving force for amplifying the immunosuppressive function of neutrophils (93). HCC cells highly express CXCL5 under hypoxic conditions, and then recruit a large number of TANs to the tumor site through the CXCL5-CXCR2 axis (94). Notably, CXCL5 not only recruits neutrophils through chemotaxis but also boosts the migratory capacity of both macrophages and Tregs (95). These cells interact in HCC-TME and jointly form a stable immunosuppressive network with TANs as an important component, ultimately disrupting the effective immune surveillance of HCC and promoting the immune escape of tumors.

## 5 Anti-tumor role of neutrophils in HCC

While the pro-tumor functions of N2 TANs dominate in established HCC, a body of evidence supports context-dependent anti-tumor activities of neutrophils, primarily mediated by N1-polarized TANs. These anti-tumor mechanisms are particularly relevant during early tumorigenesis or under specific therapeutic interventions that reprogram neutrophil function.

### 5.1 N1 TANs activating CD8+ T cells

CD8+ T cells are classic anti-tumor effector cells capable of directly recognizing and eliminating cancer cells. The anti-tumor function of N1 TANs is largely achieved through its activation and synergy with CD8+ T cells. While only a small proportion (4% to 10%) of N1 TANs can interact with tumor cells and exhibit anti-tumor activity at specific time points, their anti-tumor effect in the HCC-TME still cannot be ignored (96). N1 TANs can capture and process tumor antigens and present them directly to CD8+ T cells, thus directly initiating cytotoxic T cells to recognize and attack tumors (22). Spatial transcriptional studies (97) showed that N1 TANs are adjacent to lymphocyte clusters in the tumor tissue in spatial distribution.This proximity of physical location implies that N1 TANs can effectively promote the activation and proliferation of T and B cells locally by secreting specific cytokines or chemokines, thereby enhancing the overall anti-tumor immune response. However, this neutrophil-driven T cell activation effect is significantly time-dependent and is mainly present in the early stages of tumorigenesis (45) With tumor progression, inhibitory signals in the microenvironment (e.g., elevated TGF-β levels) prevail, driving the polarization of N1 TANs toward N2 TANs. Consequently, the T cell-activating capacity of these cells is compromised, and they may even switch to inhibiting T cell function. Therefore, the transformation of neutrophils from “anti-cancer guardians” to “accomplices in promoting cancer” stands out as a key feature of immune escape in liver cancer.

### 5.2 Neutrophil-mediated anti-tumor immune activation

Neutrophils can interact with CD47-SIRPα to mediate antibody-dependentcell-mediated cytotoxicity (ADCC), directly recognizing and phagocytosing cancer cells (98); Furthermore, they can kill tumor cells by releasing a variety of cytokines and enzymes, such as ROS, NO, and elastase. For example, by secreting NE to hydrolyse CD95 and release its dead domain, they can specifically eliminate cancer cells through interaction with histone H1 isomers (99). They kill tumor cells by releasing hepatocyte growth factor(HGF)/mesenchymal-epithelial transitionfactor (MET)-dependent NO (100).

## 6 Prognosis and therapy response of neutrophils in HCC

### 6.1 Role as a prognostic marker for HCC

A growing number of studies have utilized the number of circulating neutrophils and the ratio of neutrophils to lymphocytes as prognostic markers to assess cancer progression. A study (101)reported that absolute neutrophil count is associated with poor relapsion-free survival and cancer-specific survival of intrahepatic cholangiocarcinoma, which can be used to predict disease recurrence. A meta-analysis (102) showed that a high baseline NLR is significantly associated with poor prognosis or recurrence of HCC, especially for patients with a high incidence rate in East Asian populations. Multiple meta-analysis results (103, 104) have shown that elevated NLR levels in HCC patients before liver tumor resection are positively correlated with the occurrence of poor prognosis.

### 6.2 Relationship with immunotherapy resistance

Neutrophils can express immune checkpoint molecules, such as PD-L1 and V-domain Igsuppressor of T cell activation (VISTA) (105, 106). It has been found that (58, 107) PD-L1^+^ TANs are significantly enriched in various human and mice tumors, including hepatocellular carcinoma, melanoma, gastric cancer, intrahepatic cholangiocarcinoma, etc. Spatial transcriptomics studies (108) indicated that neutrophils are one of the main cell types expressing PD-L1 in intrahepatic cholangiocarcinoma. Specifically, PD-L1+ TANs are mainly localized near CD8+ T cells in the tumor margin and peritumoral tissues, acting as primary immune cells that suppress CD8+ T cell function. Infiltrating T cells, by binding to their ligands through PD-1 on their surface, exert an immune-negative regulatory effect. They thus inhibit the activation of T cells, cause the cells to stagnate in the quiescent phase of the cell cycle or the pre-DNA synthesis phase, render immune cells dysfunctional or even apoptotic, and participate in the immune escape of tumors, thereby promoting tumor metastasis and progression (109). Through the negative regulatory effect of PD-L1, they can inhibit CD8+ T cell function, lead to anti-PD-1/PD-L1 therapy resistance, and promote tumor development (110, 111). Meanwhile, NETs can activate the TLR4/9 pathway in dendritic cells (DCs) and macrophages, which in turn induces the upregulation of PD-L1 on these cells. Furthermore, within the inflammatory context that NETs contribute to, IFN-γ is produced by cytotoxic T cells and NK cells. This IFN- γ then acts on tumor cells and various immune cells in the tumor microenvironment, resulting in a broad upregulation of PD-L1. In summary, neutrophils directly interface with the PD-L1 immune checkpoint pathway and IFN responses primarily through two mechanisms: the self-expression of PD-L1 upon stimulation, and the perception of IFN-γ signals, which directly stimulates them to upregulate their own PD-L1 expression, thereby amplifying the immunosuppressive feedback loop (112). Therefore, elucidating the precise relationship and regulatory mechanisms between neutrophils and PD-L1 is critical for understanding the fundamental mechanisms of tumor immune escape and for developing innovative immunotherapy strategies, particularly those targeting neutrophil-mediated resistance to checkpoint blockade.

### 6.3 The role of NETs in the diagnosis and prognosis of HCC

Given the central role of NETs in the progression of HCC, their clinical translational value has become increasingly evident. A study demonstrated elevated NETs levels in both the blood and tumor tissues of HCC patients, with circulating NETs showing a strong correlation with TNM stage and the degree of hepatic dysfunction (80). A risk model constructed using NETs-related genes (HMOX1, MMP-9, TNFRSF4, MMP12, FLT3) effectively stratified HCC patients into high- and low-risk groups, with 1/3/5 year survival prediction AUC values reaching approximately 0.70. The high-risk group exhibited characteristics of an immunosuppressive microenvironment, including upregulation of immune checkpoint genes (PD-L1, CTLA-4), indicating the reliable diagnostic value of NETs markers for prognostic stratification and immunotherapy screening in HCC (113). Postoperative recurrence is one of the main reason for the poor prognosis and death of liver transplant recipients (114). Studies (115) indicated that the serum NETs levels are significantly increased in patients after liver transplantation. Meanwhile, NETs regulate the translocation of inflammatory mediators HMGB1 and M1 polarization of Kupffer cells in acute liver transplantation rejection, and even lead to acute graft rejection after liver transplantation. Therefore, the level of NETs, especially postoperative dynamic monitoring, may have a better predictive value for recurrence than the traditional AFP.

## 7 Therapeutic strategies targeting neutrophils

### 7.1 Inhibiting TANs recruitment to enhance immunotherapy efficacy

Preclinical studies have increasingly focused on disrupting pro-tumor TAN recruitment as a therapeutic strategy (116). In murine NASH-HCC models resistant to immune checkpoint inhibition, CXCR2 antagonism combined with anti-PD-1 therapy demonstrated reduced tumor burden and extended survival, accompanied by reprogramming of TANs from a protumor to anti-tumor phenotype (117). This reprogramming was characterized by the formation of granzyme B-enriched immune clusters containing CD8+ T cells and activated XCR1^+^ dendritic cells, providing mechanistic insight into the synergistic anti-tumor effects observed.

Translation to human HCC has faced significant challenges. A Phase I/II trial evaluating the CXCR2 inhibitor AZD5069 in combination with the anti-PD-L1 antibody durvalumab in advanced HCC, was terminated following discontinuation of AZD5069 development by the manufacturer (118). Importantly, no efficacy or safety results from this trial have been published in the peer-reviewed literature, precluding assessment of clinical activity or survival outcomes.Despite these setbacks, the therapeutic rationale remains compelling. Preclinical data demonstrate that CXCR2 inhibition functions not by simply reducing neutrophil numbers, but by selectively reprogramming TANs to an anti-tumor phenotype while preserving systemic neutrophil function—a critical consideration in HCC patients with underlying cirrhosis who are susceptible to infections (117). This mechanistic insight suggests that biomarker-driven patient selection based on TAN phenotype and tumor microenvironment characteristics may be essential for future clinical development. While AZD5069 development was discontinued, other agents remain in clinical development. SX-682, a dual CXCR1/2 antagonist, is being evaluated in combination with Nivolumab as a maintenance therapy in patients with metastatic pancreatic ductal adenocarcinoma (NCT04477343) (119), and navarixin, another CXCR 2 inhibitor, has demonstrated manageable safety profiles in Phase II studies across multiple cancer types (120). Recent mechanistic studies using single-cell RNA sequencing have further elucidated the IL-8/CXCR2 pathway in HCC immunotherapy resistance (121). These findings provide additional mechanistic support for targeting the CXCR2 axis in HCC.

### 7.2 Inhibiting the conversion between N1 and N2 phenotypes

As discussed in Section 2.2.2, TGF-β signaling is a key driver of N1→N2 TAN conversion within the HCC microenvironment. Therefore, therapeutic blockade of the TGF-β pathway represents a rational strategy to prevent or reverse pro-tumor neutrophil polarization.The TGF-β receptor I kinase inhibitor Galunisertib (LY2157299) is among the most extensively investigated therapeutic agents. In a Phase II study involving patients with advanced HCC, galunisertib (administered at 150 mg twice daily for 14 days, followed by a 14-day rest period) showed encouraging survival benefits and an acceptable safety profile, with a median time to tumor progression of 4.1 months and a median overall survival of 18.8 months (122). And in another clinical trial, the survival period of the experimental group with TGF-β inhibitor is longer than that of the control group, indicates this improvement may be related to the transformation of TAN phenotype (123). With the accumulation of clinical evidence on TGF-β inhibitors and the emergence of next-generation highly selective preparations, the sequential strategy of “first reshaping the TANs phenotype and then combining immunotherapy/targeted therapy” is expected to become one of the core solutions to break the immunosuppressive microenvironment of HCC and improve long-term efficacy.

### 7.3 Therapeutic strategies for targeting NETs

NETs play an important role in promoting liver inflammation and tumor growth, and inhibiting the formation of NETs can effectively alleviate the disease. In the STAM mouse model, inhibition of NETs expression levels reduces the inflammatory pattern in the liver, leading to the development of smaller tumors (124). In addition, inhibiting the formation of NETs can reduce the level of T-reg and weaken the inhibitory effect on effector T cells, thereby improving the immune surveillance effect. Besides, both the number and diameter of liver tumors in mice decrease (92). Cheng et al. (125) disrupted the structure of NETs through a hydrogel drug delivery system and found that inhibiting the expression of NETs can prevent the recurrence and metastasis of HCC after hepatectomy or liver transplantation. Direct destruction of NETs by DNase 1, combined with the anti-inflammatory drugs aspirin or hydroxychloroquine, effectively attenuates HCC metastasis in the mouse model (80). This combination strategy provides a feasible drug basis for clinical intervention. Lenvatinib, a multi-target tyrosine kinase inhibitor (TKI), is an emerging first-line therapy for HCC (126). From a mechanism perspective, lenvatinib promotes the expression and secretion of IL-33 in HCC cells, thereby triggering the formation of NETs. DNase I enhances the efficacy of lenvatinib treatment in the HCC mouse model by eliminating NETs. Blocking PAD4 or inhibiting CG can reduce the formation of NETs, while weakening the invasion and metastasis of HCC cells (127). The selective PAD4 inhibitor GSK484 can significantly inhibit NETosis in multiple tumor models and continuously improve the response of lenvatinib to HCC treatment (128). However, the role of PAD4 in gene stability makes the potential safety risks of long-term inhibition still need to be further evaluated. The optimal clinical application of NETs-targeted therapies in HCC requires careful consideration of disease stage and treatment timing. Emerging evidence suggests distinct therapeutic windows for NETs intervention strategies. For patients with early-stage HCC undergoing curative-intent therapies such as surgical resection or liver transplantation, the perioperative period represents a critical window for NETs-targeted intervention. Studies have demonstrated that NETs promote postoperative recurrence and metastasis following hepatectomy (79, 126), suggesting that prophylactic NETs inhibition during the perioperative period (approximately 1–2 weeks before and 4–6 weeks after surgery) may reduce the risk of early recurrence. This approach is particularly relevant given that the 5-year postoperative recurrence rate for early-stage HCC remains as high as 40-70% (129, 130).

In the context of advanced or unresectable HCC, NETs-targeted therapy may be most beneficial when combined with systemic treatments such as lenvatinib or immunotherapy. Recent studies have revealed that lenvatinib paradoxically induces NET formation through the NDUFA4L2/IL33 pathway, contributing to treatment resistance (128). This suggests that concurrent administration of NET inhibitors (such as DNase I or PAD4 inhibitors) with lenvatinib from the initiation of treatment may prevent the development of resistance and improve therapeutic outcomes. Similarly, NETs have been implicated in resistance to immune checkpoint inhibitors by creating an immunosuppressive tumor microenvironment and promoting T cell exhaustion (131, 132).Therefore, combination strategies targeting NETs alongside immunotherapy may be particularly valuable in patients with high baseline NETs levels or in those showing signs of primary resistance to checkpoint inhibitors.

The safety profile of NETs-targeted approaches varies depending on the specific therapeutic strategy. DNase I, which degrades the DNA backbone of NETs, has been used clinically for decades in patients with cystic fibrosis to reduce mucus viscosity, with a well-established safety profile (133). However, systemic DNase I administration in cancer patients may carry risks of increased susceptibility to bacterial infections, particularly in those with pre-existing immunocompromised states or during periods of myelosuppression following chemotherapy. The short half-life of recombinant DNase I necessitates frequent dosing, which may limit its practical application in chronic oncology settings.PAD4 inhibitors represent a more targeted approach to NET inhibition, as PAD4-mediated histone citrullination is essential for chromatin decondensation during NETs formation. The selective PAD4 inhibitor GSK484 has demonstrated efficacy in preclinical cancer models, significantly reducing NET formation and improving response to lenvatinib in HCC (128, 134). However, PAD4 plays important roles beyond NETs formation, including regulation of gene expression and maintenance of genomic stability (135). Long-term PAD4 inhibition may therefore carry theoretical risks of altered gene expression patterns and potential genotoxicity. The development of next-generation PAD4 inhibitors with improved isoform specificity, such as BMS-P5, may help mitigate off-target effects, though comprehensive safety assessments remain necessary (136).

In conclusion, while significant challenges remain in the clinical translation of NETs-targeted therapies for HCC, the growing body of preclinical evidence and the development of innovative delivery strategies provide a strong foundation for future clinical investigation. Careful attention to clinical timing, safety considerations, and localized delivery approaches will be essential for realizing the therapeutic potential of NETs-targeted interventions in HCC management.

### 7.4 Neutrophils and tumor metabolic reprogramming: the role of neutrophil-derived extracellular vesicles

NDEVs are important carriers for metabolic communication between tumor cells and stromal cells, capable of transporting metabolic enzymes, metabolites, mirnas, etc., and directly reshaping the metabolic state of the receiving cells (137). Studies have revealed that neutrophil-derived extracellular vesicles and NETs residues are key mediators connecting inflammation and metabolism. NDEVs can directly ‘deliver’ complete glycolytic enzymes (e.g., PKM2 and GAPDH) to tumor cells, thereby forcibly enhancing their aerobic glycolytic flux (138). Meanwhile, NETs activate TLR signals on the surface of tumor cells through their protein components (e.g., NE), thereby upregulating the expression of glutaminase-1 and driving the ‘glutamine addiction’ of tumor cells to meet the biosynthetic requirements for their rapid proliferation (138).

Neutrophil-derived metabolic reprogramming offers actionable biomarkers and therapeutic targets that bridge innate immunity and cancer metabolism in HCC. Circulating NETs can be reliably quantified in HCC patient plasma using validated ELISA. Specifically, H3Cit-DNA (citrullinated histone H3-DNA) and MPO-DNA (myeloperoxidase-DNA) complexes serve as specific NET biomarkers, with H3Cit-DNA levels exceeding 200 ng/mL indicating high NET activity and correlating with advanced liver dysfunction (Child-Pugh B/C) and elevated inflammatory markers (139). Furthermore, NDEVs containing PKM2 can be isolated from patient plasma through size-exclusion chromatography. PKM2-containing ectosomes from HCC cells induce macrophage differentiation and promote tumor growth, with PKM2 serving as both a metabolic biomarker and a functional mediator of tumor microenvironment remodeling (140, 141). HCC patients with elevated plasma H3Cit-DNA levels (> 200 ng/mL) or high MPO-DNA complexes represent a distinct molecular subgroup characterized by inflammation-driven pathology, impaired liver function (Child-Pugh score ≥ 7), and heightened systemic inflammation (C-reactive protein elevation) (139). This stratification is clinically relevant, as preclinical studies demonstrate that NETs inhibition significantly reduces HCC tumor growth in nonalcoholic steatohepatitis-associated models (124). Therefore, patients with high NET signatures should be prioritized for neutrophil-targeted clinical trials, as they may derive maximal benefit from therapies targeting the NET-cancer axis.

PKM2-mediated glycolytic reprogramming through NDEVs suggests that tumors with high extracellular vesicle activity may exhibit heightened sensitivity to glycolysis inhibitors. Lactate dehydrogenase A (LDHA) inhibitors, including FX11 and GNE-140, effectively disrupt HCC glycolysis by blocking the conversion of pyruvate to lactate, leading to ATP depletion, proliferation arrest, and apoptosis (142). Additionally, DNase I combined with anti-inflammatory agents (aspirin/hydroxychloroquine) effectively reduces HCC metastasis by disrupting both NETs-mediated inflammation and tumor cell adhesion (80). This dual-pronged approach—simultaneously targeting NETs-mediated inflammatory signaling and tumor metabolic reprogramming—represents a rational combination strategy to disrupt the self-reinforcing oncogenic loop driving HCC progression.

Therefore, neutrophils function as “metabolic regulators” that package inflammatory signals and metabolic instructions into extracellular vesicles and NETs, creating a self-reinforcing oncogenic loop that coordinates chronic inflammation with tumor metabolic reprogramming. Targeting this axis through biomarker-guided patient stratification and rational combination therapies offers a promising translational strategy for HCC treatment.

### 7.5 Prospects of combined strategies with immunotherapy

In the immune system, immune checkpoints have regulatory functions that maintain immune tolerance and regulate the intensity and duration of immune responses in peripheral tissues. Immunotherapy for HCC using immune checkpoint inhibitors (ICI) has emerged as a highly promising treatment approach (143). Several exploratory studies of ICI have been conducted in the field of liver cancer. Following the release of data from the CheckMate 040, KEYNOTE-240, KEYNOTE-224, and SHR-1210 trials, pembrolizumab (PD-1 monoclonal antibody) and atezolizumab (PD-L1 monoclonal antibody) have been sequentially included in several guidelines and endorsed as clinical treatment choices for liver cancer. Studies have shown (144)that in mouse HCC model, cabozantinib combined with anti-PD-1 antibody can promote neutrophil-mediated immune responses, thereby enhancing the anti-tumor effect of the drug.

## 8 Beyond the target: direct development of neutrophils as therapeutic agents

In addition to targeting neutrophil infiltration or function, an emerging therapeutic paradigm is to develop neutrophils themselves directly as an intelligent therapeutic tool for HCC. Taking advantage of the natural tendency of neutrophils towards inflammatory and tumor sites, especially in the HCC microenvironment rich in chemokines such as IL-8, the following strategies show great potential.

### 8.1 As a targeted drug delivery carrier

By taking advantage of the natural chemotaxis of neutrophils, biomimetic nanocarriers can be constructed to achieve precise drug delivery to HCC. For instance, Kang et al. (145)developed nanoparticles coated with neutrophil cell membranes, which perfectly inherited the targeting ability of neutrophils to inflammatory sites and their immune escape characteristics. In the HCC lung metastasis model, such particles loaded with chemotherapy drugs significantly enhanced the drug efficacy and inhibited metastasis. This provides strong evidence for the use of neutrophil components in the treatment of HCC, especially advanced HCC with inflammatory features.

### 8.2 Adoption and reinfusion of polarized neutrophils

Isolating neutrophils from the patient’s body, reprogramming them from the potential N2 phenotype to the anti-tumor N1 phenotype *in vitro*, and then reinfusing them is an individualized treatment strategy. Studies have shown that pretreatment of neutrophils with Toll-like receptor agonists (e.g., R848) or IFN-β can polarize them into an N1 state that produces high levels of pro-inflammatory cytokines and has stronger cytotoxicity, thereby inhibiting progression in tumor models (19). This provides a direct approach to reversing the immunosuppressive microenvironment of HCC.

### 8.3 Engineered CAR-neutrophils

Despite the huge challenges, endowing neutrophils with specific targeting capabilities is a cutting-edge direction. Chang et al. (146) reported that CAR-neutrophils expressing chimeric antigen receptors targeting EGFR demonstrated effective anti-tumor activity in glioma models. Given that specific antigens such as GPC-3 and AFP are often expressed in HCC, the development of CAR-neutrophils targeting these antigens is expected to make them “live drugs” that directly kill HCC cells. Although it is still in the exploration stage at present, considering the powerful phagocytic and killing abilities of neutrophils, the development of CAR-neutrophils targeting HCC-related antigens may become an exciting research direction in the future.

## 9 Summary and prospects

This review systematically synthesizes the multifaceted roles of neutrophils in the pathogenesis and progression of HCC, along with their potential therapeutic implications. Targeting neutrophils is not intended to replace existing standard therapies, but rather to expand and enhance our therapeutic arsenal. As outlined in perspectives on future cancer treatment, success will inevitably depend on rational combination strategies (147).Accordingly, neutrophil-targeting approaches, including CXCR2 inhibitors, TGF-β inhibitors, and NETs degradation therapies, represent highly promising synergistic partners for established treatments. With several neutrophil-modulating therapies currently in clinical trials for HCC and other malignancies, the therapeutic landscape continues to evolve rapidly.Looking ahead, we are optimistic that with deeper understanding of neutrophil biology and development of tools capable of precisely targeting specific neutrophil subsets or functional states, neutrophil-directed strategies will find their niche in the evolving landscape of precision combination therapy for HCC.
